# Society coexisting with COVID-19

**DOI:** 10.1017/ice.2020.130

**Published:** 2020-04-13

**Authors:** Kazuhiro Tanabe

**Affiliations:** 1 Medical Solution Promotion Department, Medical Solution Segment, LSI Medience Corporation, Tokyo, Japan; 2 Research Supporting Department, Kyushu Pro Search, Fukuoka, Japan

*To the Editor—*Following the first case reports of coronavirus disease 2019 (COVID-19) in Wuhan, China in December 2019, the cumulative number of cases reported skyrocketed to 800,000 by the beginning of April 2020.^1^ This pandemic has yielded an accurate and unprecedented global epidemiological record that includes the correct number of cases and deaths reported in >200 countries. This record provides clues as to how we can address this invisible enemy.

We calculated spreading speed (SS1000) of COVID-19 in 15 countries using figures provided by the World Health Organization (WHO).^1^ The SS1000 is defined as the period in which the total number of cases increases from 100 to 1,000. In Italy, the total number of cases reached 100 on February 24, and subsequently reached 1,000 on March 1, therefore Italy’s SS1000 was 6 days. Observing the SS1000’s of 15 countries (Fig. 1), Japan’s SS1000 stands out because it is distinctly longer than those of the other 14 countries. Despite the SS1000s of most countries being <12 days, Japan’s SSA was 28 days.

**Fig. 1.**
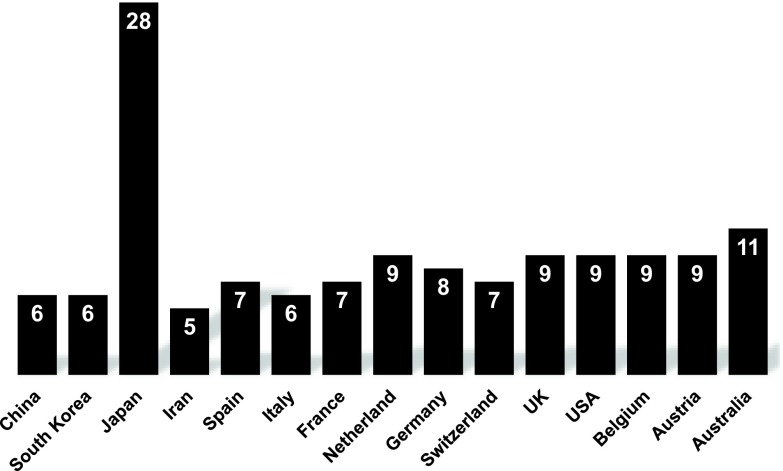
Spread speed of COVID-19 (ie, SS1000) in 15 countries.

These figures were provided by the World Health Organization.^1^ The SS1000 is defined as the period taken for the total number of cases to increase from 100 to 1,000.

The reasons for Japan’s relatively long SS1000 have been a source of debate. Some have attributed it to the Japanese government’s rapid and appropriate response, such as the closure of all schools on March 2, when <300 cases had been confirmed within the country. Others have attributed it to the high level of discipline exhibited by Japanese people, for example, full-time wearing of face masks, frequent hand washing, and not talking in crowded trains.

However, considering Japan’s high population density and its rapidly aging population, the COVID-19 situation in Japan may be more serious than it currently appears. Among various opinions that have been expressed, we are of the opinion that the limited number of tests (26,607 total tests conducted by March 30)^2^ compared to other countries (eg, South Korea with 395,194 tests)^3^ may be attributed to Japan’s slow SS1000 and the low number of confirmed cases.

Why is number of tests conducted in Japan so small? First, the private practice system in Japan requires a person to have a doctor’s diagnosis in order to access testing. Second, the government announced a strict policy on February 17, 2020, that requires people to visit a doctor only if they have 4 days of continuous fever (>37.5°C). This criterion constitutes a major barrier that prevents people with mildly symptomatic COVID-19 from accessing testing. Therefore, whether intentional or not, the number of confirmed COVID-19 cases in Japan may have been greatly underestimated compared with that of other countries.

On the other hand, if Japan has a large number of asymptomatic carriers, why has the reported number of COVID-19–related deaths (ie, 59 as of March 31, 2020) has remained so low compared with other countries such as Italy (11,591), Spain (7,340), and France (3,024)?^1^ First, by limiting the number of patients, Japanese medical staff could concentrate on only patients with severe symptoms, which reduced the risk of infection to medical staff themselves. Also, the number of COVID-19 deaths may have been underestimated. If we hypothetically assume that there have been 500 COVID-19–related deaths in Japan to date (ie, >10 times the confirmed number of deaths), the increase may not have been noticed because these deaths are hidden among the 100,000 pneumonia-related deaths that occur every year in Japan.^4^

We should appreciate that Japanese have not faced any social panic or medical collapse due to the COVID-19 epidemic and that Japanese society is functioning relatively normally despite partially limited (eg, 1 month) school closures. Moreover, some functions of society have started to recover. Whether intentional or not, Japan’s choice to limit the opportunity of testing has helped to prevent social disruption.

How should we face COVID-19? We must ascertain the true case fatality rate (CFR), and to do this, we need to determine the true number of cases, including not only severe symptomatic cases but also asymptomatic and mildly symptomatic cases. In Japan, the government conducted screening of passengers on the Diamond Princess cruise ship, and these figures can be considered “real” confirmed cases. According to Japan’s Ministry of Health, Labor and Welfare, the total number of COVID-19 cases was 712 with 10 related deaths,^5^ for a CFR of 1.4%. Considering the elderly passenger population, and their long-term confinement in the ship under poor conditions, the actual CFR may be less than the calculated value; however, it is currently the most reliable estimate of the COVID-19 CFR.

Admittedly, 1.4% CFR is too high to ignore, but in my opinion, it is an acceptable level at which we can coexist. Japanese policies were not only successful in containing the epidemic but also helped to avoid social chaos, which tell us that excessive responses do not always have expected results. Limiting society functions, such as strict lockdown, is often accompanied by major adverse effects among the socially vulnerable, including elderly people or the patients suffering from the other diseases, and it may result in deaths from other causes. Therefore, Japan’s choices in facing an unprecedented and frightening epidemic should be well considered.

## References

[1] Coronavirus disease (COVID-19) Pandemic, April 2, 2020. World Health Organization website. https://www.who.int/emergencies/diseases/novel-coronavirus-2019 Published April 2, 2020. Accessed April 15, 2020.

[2] Coronavirus disease (COVID-19) situation report in Japan, March 30, 2020. Toyo Keizai website. https://toyokeizai.net/sp/visual/tko/covid19/en.html Published March 30, 2020. Accessed April 15, 2020.

[3] Number of coronavirus (COVID-19) confirmed, recovered, and test cases in South Korea, March 28, 2020. Statista website. https://www.statista.com/statistics/1095848/south-korea-confirmed-and-suspected-coronavirus-cases/ Published March 28, 2020. Accessed April 15, 2020.

[4] Population survey report, March 21, 2020. Japan Ministry of Health, Labour, and Welfare website. https://www.mhlw.go.jp/toukei/saikin/hw/jinkou/suii09/deth8.html Published March 21, 2020. Accessed April 15, 2020.

[5] About Coronavirus Disease 2019 (COVID-19) March 26, 2020. Japan Ministry of Health, Labour and Welfare website. https://www.mhlw.go.jp/stf/seisakunitsuite/bunya/newpage_00032.html Published March 26, 2020. Accessed April 15, 2020.

